# AP-2δ Is a Crucial Transcriptional Regulator of the Posterior Midbrain

**DOI:** 10.1371/journal.pone.0023483

**Published:** 2011-08-09

**Authors:** Katrin Hesse, Kristina Vaupel, Simone Kurt, Reinhard Buettner, Jutta Kirfel, Markus Moser

**Affiliations:** 1 Institute of Pathology, University of Bonn, Bonn, Germany; 2 Max-Planck-Institute of Biochemistry, Martinsried, Germany; 3 Institute of Neurobiology, University Ulm, Ulm, Germany; 4 Institute of Pathology, University of Koeln, Koeln, Germany; University of Memphis, United States of America

## Abstract

Ap-2 transcription factors comprise a family of 5 closely related sequence-specific DNA binding proteins that play pivotal and non-redundant roles in embryonic organogenesis. To investigate the function of Ap-2δ, wδe analyzed its expression during embryogenesis and generated Ap-2δ-deficient mice. In line with the specific expression pattern of Ap-2δ in the mesencephalic tectum and the dorsal midbrain, Ap-2δ-deficient mice failed to maintain the colliculus inferior, a derivative of the dorsal midbrain, as a consequence of increased apoptotic cell death. To identify specific Ap-2δ target genes in cells of the developing dorsal midbrain, we performed whole genome analysis of cDNA expression levels. This approach identified a set of 12 putative target genes being expressed in the developing midbrain, including the transcription factors Pitx2, Mef2c, Bhlhb4 and Pou4f3. Using chromatin immunoprecipitation (CHIP) we showed that some of these genes are direct targets of Ap-2δ. Consistently, we demonstrate that Ap-2δ occupies and activates the Pou4f3 and Bhlhb4 promoters. In addition, known Pou4f3 target genes were downregulated in the posterior midbrain of Ap-2δ-deficient mice. Despite the absence of a central part of the auditory pathway, the presence of neuronal responses to sounds in the neocortex of Ap-2δ-deficient mice indicates that auditory information from the brainstem still reaches the neocortex. In summary, our data define Ap-2δ as an important transcription factor, specifying gene expression patterns required for the development of the posterior midbrain.

## Introduction

Ap-2 transcription factors are closely related, sequence-specific DNA-binding proteins that play pivotal roles in regulating gene expression during development, differentiation, and tumorigenesis [1]. In mammals, five Ap-2 genes (*Tcfap2a* to *e*) exist, which encode for the Ap-2α to ε proteins. Ap-2α, Ap-2β and Ap-2γ show partially overlapping expression patterns in neural crest cells, the central and peripheral nervous system, the facial mesenchyme, the limbs, various epithelia of the developing embryo, and the extraembryonic trophectoderm [2], [3], [4]. In contrast, the more recently characterized Ap-2 transcription factors, Ap-2δ and Ap-2ε, show a more restricted expression pattern and are almost exclusively expressed in the central nervous system. Ap-2ε was mainly found in the developing olfactory bulb, the vomeronasal epithelium [5], [6], and hypertrophic chondrocytes [7], whereas Ap-2δ is predominantly expressed in the midbrain. In addition, Ap-2δ is expressed at lower levels in the diencephalon, forebrain, spinal cord and the retina and for a short period in the developing heart [8].

Until now all Ap-2 isoforms, except for *Tcfap2d,* have been inactivated in mouse and their phenotypes indicate individual and non-redundant functions during mouse development. Mice lacking Ap-2α die perinatally with neural tube, limb mesenchyme and craniofacial defects [9], [10], [11]. Ap-2β knockout mice develop polycystic kidney disease and terminal renal failure and die shortly after birth [12], [13]. Ap-2γ controls proliferation and differentiation of extraembryonic trophectodermal cells and deficiency results in early embryonic lethality due to defective implantation and nutrition of the developing embryo [14], [15]. A recent study on Ap-2ε deficient mice revealed disorganized neuronal lamination of the olfactory bulb [16].

Ap-2 proteins have been reported to form homodimers as well as heterodimers that bind to highly conserved GC-rich consensus sequence present in many cellular and viral promoters and enhancers. An *in vitro* binding site selection assay identified GCCN_3/4_GGC and GCCN_3/4_GGG as the preferred sequence motifs bound by Ap-2 transcription factors [17]. The Ap-2 family members share a highly conserved basic DNA-binding domain and a carboxy-terminal helix-span-helix motif that mediates DNA binding and dimerization [18], [19]. Ap-2δ is considered the most divergent family member because its transactivation domain lacks certain amino acids, which are thought to play a critical role for its transcriptional activity. Based on this observation it is believed that Ap-2δ interacts with a different set of coactivators and target genes. To address the function of Ap-2δ *in vivo*, we disrupted the *Tcfap2d* gene in mice. Ap-2δ-deficient mice are viable but lack parts of the posterior midbrain due to massive apoptotic cell death, which initiates at the end of embryogenesis. An Ap-2δ target gene screen from the posterior midbrain showed that several transcription factors are regulated by Ap-2δ suggesting that the normal differentiation program of this structure is disturbed in the absence of Ap-2δ leading to programmed cell death. Surprisingly, despite of the absence of the colliculus inferior in Ap-2δ-deficient mice neuronal responses to sounds were recorded in the neocortex indicating that a different auditory route towards the auditory cortex is used in Ap-2δ-knockout mice.

## Materials and Methods

### ES cell culture and generation of Ap-2δ-null mice

**Figure 1:**
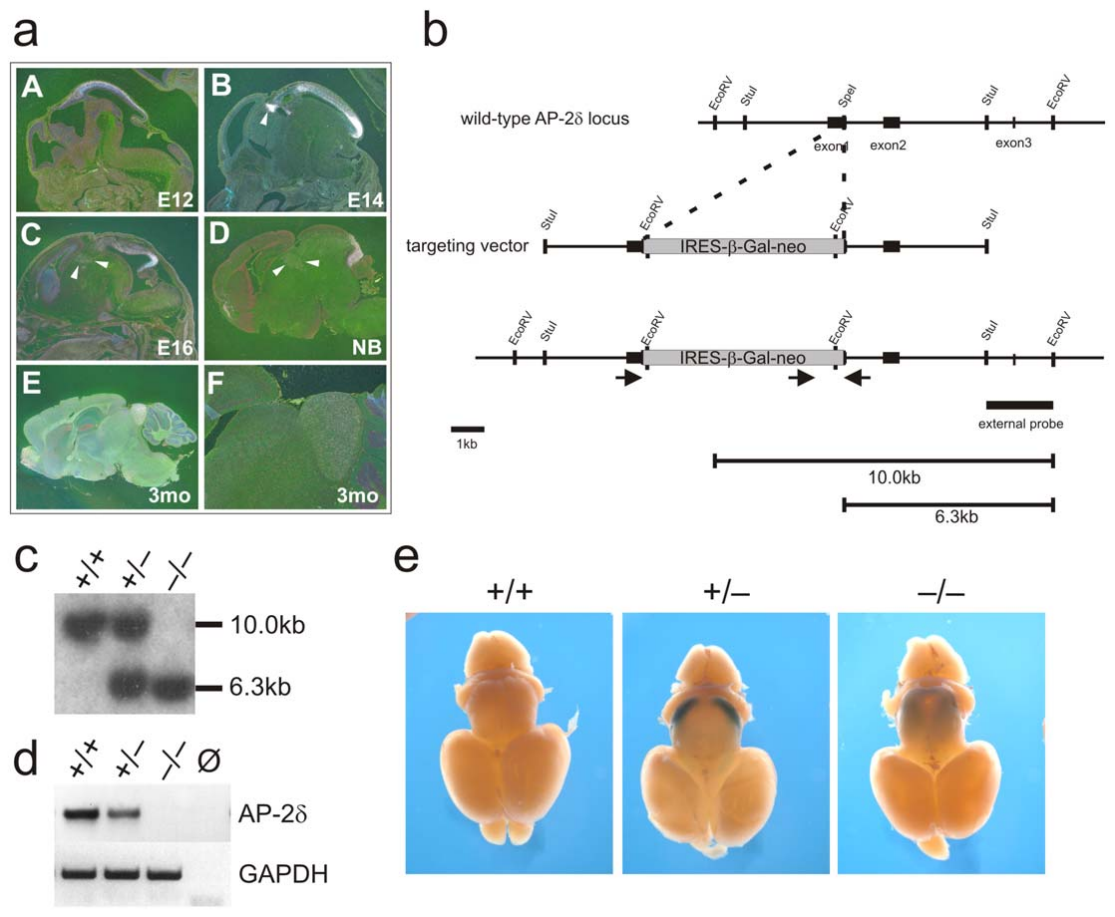
Generation and analysis of Ap-2δ-defecient mice. (a) Radioactive in situ hybridization with an Ap-2δ-specific probe on sagittal sections of either whole embryos (E12-E16; A–C) or isolated brains (D–F) of newborns (NB; D) and three months old mice (3 mo; E, F). Ap-2δ is mainly expressed in the posterior mesencephalon (A–F) and weakly in the dorsal thalamus (A–D; white arrows B–D) (b) Schematic representation of the partial *Tcfap2d* gene locus and the targeting construct. Black boxes indicate *Tcfap2d* exons 1 through 3 and the black box indicates the probe used for Southern hybridization which recognizes the 10 kb wild-type and the 6.3 kb mutant *Tcfap2d* EcoRV fragment. (c) Southern blot analysis of tail biopsy DNAs from the progeny of mating heterozygotes. (d, e) Confirmation of *Tcfap2d* gene deletion by RT-PCR analysis on cDNA of E17 posterior midbrains extracted from wild-type (+/+), heterozygous (+/−) and Ap-2δ-deficient mice (−/−) (d) and by X-Gal staining (e) on brains of newborns. In brains from heterozygous animals, Ap-2δ promoter activity can be detected in the posterior midbrain (arrowhead). In contrast, in Ap-2δ-deficient animals the LacZ staining is diffusely spread throughout the midbrain (arrowhead) (e).

The full-length Ap-2δ cDNA was used to screen a Sv129 PAC library. A 10.2 kb EcoRV fragment containing exons 1 to 3 of the *Tcfap2d* gene was subcloned into pBluescript II SK (-) (Stratagene). The targeting vector was designed to disrupt exon 1 by an inserted IRES-β-Gal-neo cassette, flanked by 3.0 kb 5′ homologous and 4.3 kb 3′ homology arms according to (13). ES cells and Ap-2δ mutant mice were genotyped by Southern blot analyses (Fig. 1c) or genomic PCR. For PCR analyses, a forward primer (5′-AATGCATCGTAAGCTTTTGGAG-3′) was used in combination with a reverse primer (5′-GGAGGAGAGTTGCCCTCCCTGCG-3′) to determine the wild-type allele, and a third primer (5′-CTGCTCTTTACTGAAGGCTCTTT-3′) to detect the null allele. Mice were kept at the MPI of Biochemistry animal facility in accordance to Bavarian animal welfare laws. Generation of Ap-2δ deficient mice does not require permission of the Bavarian animal protection law. The generation of Ap-2δ deficient mice was carried out in strict accordance with all German (e.g. German Animal Welfare Act) and EU (e.g. Directive 86/609/EEC) applicable laws and regulations concerning care and use of laboratory animals. The Max Planck Institute of Biochemistry has a licence for breeding and housing of laboratory animals (No. 5.1-568 - rural districts office). This includes the generation of knockout mice by ES cells injection. For this kind of experiments no separate licence or an approval of an ethics committee is required in the District Upper of Bavaria. All animals used were bred for scientific purposes.

The Max Planck Institute of Biochemistry is registered at NIH and has a PHS Approved Animal Welfare Assurance from the Office of Laboratory Animal Welfare: #A5132-01. (see:http://grants.nih.gov/grants/olaw/assurance/500index.htm? Country = GM#GridTop)

### In situ hybridization, whole mount in situ hybridization, X-Gal staining, immunohistochemistry and immunofluorescence staining

In situ hybridizations and whole-mount in situ hybridizations were performed essentially as previously described [20], [21]. For X-Gal staining, brains were isolated, washed in 100 mM sodium-phosphate buffer pH 7.3 and fixed in 0.2% glutaraldehyde, 5 mM EGTA, 2 mM MgCl_2_ in 100 mM sodium-phosphate buffer pH 7.3 for 30 min. Upon washing in 2 mM MgCl_2_, 0.02% NP-40 in sodium-phosphate buffer pH 7.3, tissues were stained in X-Gal staining solution (1 mg/ml X-Gal, 5 mM K_4_Fe(CN)_6_, 5 mM K_3_Fe(CN)_6_ in wash buffer) for several hours at 37°C. Immunohistochemical analysis with the Ap-2δ specific antiserum was performed exactly as described [6]. Immunofluorescence staining was performed using secondary antibodies from Molecular Probes/Invitrogen.

### RNA isolation, Affymetrix microarray procedures and qRT-PCR

Micro-dissected posterior midbrains from 15 Ap-2δ−/− and wild-type E15 embryos each were pooled and total RNA was purified using the RNeasyMini-kit from Qiagen as specified by the manufacturer. For microarray analyses, we used the Affymetrix GeneChip platform employing a standard protocol for sample preparation and microarray hybridization. Total RNA (2.5 µg) was converted into biotinylated cRNA according to the Affymetrix standard protocol version 2, purified, fragmented and hybridized to Affimetrix GeneChip Mouse Genome xY 2. =  ArraysHG-U133Plus_2.0 microarrays (Affymetrix Inc.). The microarray data has been released into the GEO-database (Accession Number GSE27296).

Gene expression was monitored by quantitative real-time PCR (Applied Biosystems). Expression values were normalized to the mean of HPRT. Primer sequences are available upon request.

### Antiserum production and Western blot analysis

An Ap-2δ specific peptide (GSQYGMHPDQRLLPG) was coupled to Imject Maleimide Activated mcKLH (Pierce) and used to immunize rabbits. Protein lysates were extracted from cells, blotted and incubated as described [22]. Dilutions of the antibodies: α-Ap-2δ 1∶5.000, α-HA (Hiss Diagnostics) 1∶5000; β-actin (Sigma-Aldrich) 1∶20.000; α-tubulin (Abcam) 1∶10.000.

### Chromatin immunoprecipitation (ChIP)

ChIP experiments were performed essentially as described [23]. Neuro2a cells were transfected with an HA-Ap-2δ expression plasmid 2 days before harvesting for ChIP. Immunoprecipitation was performed with specific antibodies to HA and IgG (Diagenode) as a negative control on protein A coupled Dynabeads (Invitrogen). Amplicons were normalized to the DNA immunoprecipitated with antibody to histone H3 (Abcam). Recovered DNA was analyzed by TaqMan real-time PCR using the following loci-specific primers: Bhlhb4 -300 bp: 5′-AGAAATGGCAGTTGAGGGG-3′, 5′-AATCAGAGGGGACCTGCTG-3′, Bhlhb4 -1.8 kB: 5′-TCTCTTCCTAGCCTCCCACA-3′, 5′-GCTGTTCAGGGGAGTCT GTC-3′, Pou4f3 -2.6 Kb: 5′-TGACCATTGCTAGTGGACCTT-3′, 5′-CCAATGCGGTTCA ACAGAC-3′. Values were displayed as percent H3 with H3 occupancy arbitrarily set to 100%.

### Cell culture and transfection

Neuro2A cells were cultured in DMEM supplemented with 10% FCS. Transient transfection assays were carried out using the standard calcium phosphate technique. Luciferase (LUC) activity was assayed as recommended by the manufacturer (Promega) in the Luminometer Centro LB 960 (Berthold). Relative light units were normalized to protein concentration using the Bradford dye assay (Bio-Rad). All experiments were repeated at least five times.

### Reporter and expression plasmids

The LUC reporter plasmid Pou4f3 −2.8 kb (BAC clone (RPCIB731H07316Q, imaGenes)) and Bhlhb4–6 kb were generated using PCR amplification followed by insertion of the PCR product into pGl3-basic (Promega). Bhlhb4–0.5 kb was generated by subcloning of Bhlhb4–6 kb. Expression plasmid pSG5-Ap-2δ was generated by excision of Ap-2δ cDNA from pCMX-Ap-2δ and insertion into pSG5 vector (Stratagene). For pSG5-HA-Ap-2δ, a cDNA fragment encoding Ap-2δ downstream of an optimal Kozak sequence and an HA tag was generated by PCR amplification using pSG5-Ap-2δ as template. All generated constructs were verified by sequencing.

### DNA binding studies

Ap-2δ was *in vitro* translated using the TNT-coupled reticulocyte extract (Promega). Ap-2δ primed lysate was incubated with approximately 0.8 ng of the ^32^P-labeled oligonucleotide probe according to Zhao et al. [8]. Subsequently, the samples were separated on 5% nondenaturing polyacrylamide gels in 0.25× TBE running buffer. Gels were dried and subjected to autoradiography at −80°C. The following oligonucleotides were used: Zhao 5-′GAACTGACCGCCTGAGGCGCGTGTGCA-3′, 5-′TGCACACGCGCCTCAGGCGG-TCAGTT-3′, Pou wt 5′-TTTCTAGGGGC-CTGAGGGAAGTAGTGG-3′, 5-‘CCACT-ACTTCCCTCAGGCCC-3’, Pou mt1 5′-TTTCTAGGGGCCAGTGGGAAGTAGTGG-3′, 5-‘CCACTACTTCCCACTGGCCC-3’, Pou mt2 5′-TTTCTAGGGGCCGTTGGG-AAGTAGTGG-3′, 5-‘CCACTACTTCCCAACGGCCC-3’. To determine the composition of various DNA-protein complexes, the following antibodies were included during incubation: α-Ap-2δ and rabbit α-IgG (Diagenode).

### Recordings of neuronal responses from neocortex

Recordings were performed in the caudal part of the temporal cortex of two anesthetized (Ketamin/Xylazin, 75 mg/kg/5 mg/kg) Ap-2δ−/− mice in an acoustically and electrically shielded recording chamber. Multiunit activity was recorded with tungsten electrodes (WPI, TM 33A10KT, 1 MΩ impedance), amplified (20,000 times), filtered (bandpass from 250–8,000 Hz), digitised (40 kHz sampling at 12-bit resolution) and stored in a multichannel recording system (Multichannel Acquisition Processor; Plexon). Electrode penetrations were made perpendicular to the cortical surface and recordings were obtained in a depth of 300–350 µm from cortical layers III-IV from at least 10 neurons from Ap-2δ−/− mice and compared with those of wildtype animals. Acoustical stimuli were digitally synthesized and controlled pure tones (MathWorks) presented in pseudorandomized series of bursts (200 ms duration, 5 ms rising and falling ramp, 600 ms inter-burst interval) of frequencies with a logarithmic, iso-octave spacing spanning 6 octaves from 1000 Hz to 64 kHz. Tone bursts were delivered via an attenuator (gPAH, gtec,) and amplifier (PMA 1060, Denon) to a loudspeaker (W06, Manger) positioned 30 cm in front of the animal's head. All tones were presented at a sound pressure level (SPL) of 70±5 dB at the ear of the animals. Significant tone-evoked responses were determined two standard deviations above spontaneous activity level.

### Statistical methods

Statistical significance was calculated using the Student's t-test. A p-value less than 0.05 was considered to be significant. (*  =  p<0.05; **  =  p<0.005; ***  =  p<0.0005)

## Results

### Generation of Ap-2δ-deficient mice

Ap-2δ is almost exclusively expressed in the central nervous system [8]. A detailed analysis of Ap-2δ expression during development and in the postnatal brain was performed as a prerequisite for the analysis of Ap-2δ mutant mice. Therefore, we visualized Ap-2δ expression on sagittal sections of mouse embryos and isolated brains of newborns and 3 months old mice, respectively, using in situ hybridization. At E12.5 and E14.5, Ap-2δ localizes to the mesencephalic tectum and the dorsal diencephalon (Fig. 1a, A–B). Already at E14.5 a more intense Ap-2δ signal becomes apparent in the posterior midbrain. This shift in Ap-2δ expression towards the posterior midbrain is even more striking at later stages of development (Fig. 1a, C) and is completed at the newborn stage (Fig. 1a, D). After birth the dorsal midbrain separates into an anterior part, the colliculus superior, and a posterior structure, the colliculus inferior. In the adult brain, Ap-2δ is predominantly expressed in the colliculus inferior (Fig. 1a, E, F). Additionally, weak Ap-2δ expression can be detected in the dorsal thalamus and the forebrain both during embryogenesis and adulthood (Fig. 1a white arrows).

To inactivate the *Tcfap2d* gene in mice we screened a PAC library with an Ap-2δ cDNA probe and isolated a 10-kb clone spanning exons 1–3 of the gene (Fig. 1b). To disrupt the Ap-2δ coding sequence we inserted a β-galactosidase-neomycin cassette into exon 1 of the *Tcfap2d* gene. Heterozygous Ap-2δ mice (Ap-2δ+/−) are fertile and show no overt phenotype. A Southern blot of wild-type, Ap-2δ+/− and Ap-2δ-deficient (Ap-2δ−/−) mice confirmed the targeted mutation of the *Tcfap2d* gene locus (Fig. 1c). Consistently, RT-PCR analysis with Ap-2δ-specific primers on RNA from posterior midbrains of E17.5 embryos verified a significant reduction in Ap-2δ expression in heterozygous mice and the complete inactivation of the *Tcfap2d* gene in Ap-2δ-deficient mice (Fig. 1d). Next, we performed whole mount LacZ stainings on brains from newborn wild-type, heterozygous and Ap-2δ deficient mice. In line with previous expression data, strong ß-galactosidase activity, which indicates high Ap-2δ promoter activity, is present in the posterior midbrain and a much weaker staining can be seen in the anterior midbrain and the diencephalon of Ap-2δ+/− mice. In contrast, LacZ staining on brains from Ap-2δ-deficient mice did not show a discrete labelling of the posterior midbrain but instead was diffusely spread throughout the entire midbrain (Fig. 1e).

### Ap-2δ-deficient mice lack the colliculus inferior

**Figure 2:**
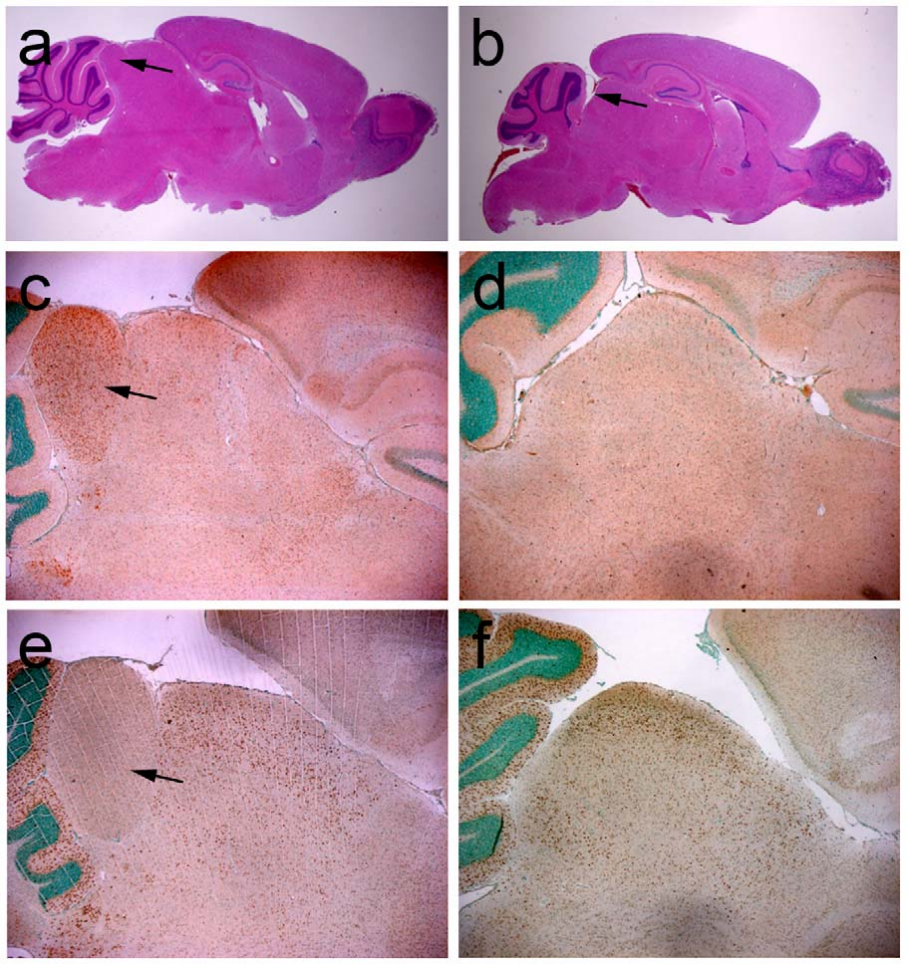
Histological analysis of wild-type and Ap-2δ-deficient mesencephalon. (a, b) Hematoxylin-eosin staining on midbrain paraffin sections of 1.5 months old mice. The arrows mark the colliculus inferior, which is absent in Ap-2δ-deficient mice. (c–f) Immunohistological analysis with Ap-2δ (c, d) and Ap-2α (e, f) -specific antibodies on sagittal sections from 3 weeks old mice midbrains. Ap-2δ expression is restricted to the colliculus inferior and, to a lesser extent, to the colliculus superior and the dorsal thalamus. Ap-2α is expressed in the colliculus superior and the cerebellum of wild-type animals. In Ap-2δ-deficient mice, the expression of Ap-2α extends throughout the entire mesencephalon.

We next wanted to know whether loss of Ap-2δ affects brain morphology. Sagittal sections through the brain of 6 weeks old Ap-2δ−/− mice showed a normal organization into forebrain, midbrain and hindbrain. However, the posterior midbrain, the colliculus inferior, was missing in Ap-2δ mutant mice (Fig. 2a,b). In order to analyse Ap-2δ function in more detail we generated an isoform-specific Ap-2δ antibody, that revealed a 50 kDa band in HepG2 cells transfected with an Ap-2δ cDNA but did not show any crossreactivity with any other Ap-2 isoform (Figure S1a). Its specificity was also confirmed by transfection experiments into NIH3T3 cells and in immunohistological stainings on sections from E13.5 embryos that revealed a nuclear staining in cells of the diencephalon and midbrain, the two major regions in the developing brain that are also labelled by whole mount in-situ hybridizations of embryos hybridized with an Ap-2δ-specific cDNA probe (Figure S1 b–d). Using Ap-2δ and Ap-2α-specific antibodies on paraffin sections of adult wild-type brains mapped Ap-2δ expression to the colliculus inferior and, to a lesser extent, to the dorsal thalamus and the colliculus superior (Fig. 2c). Immunohistochemical staining of sagittal brain sections from Ap-2δ−/− mice revealed no signal (Fig. 2d). In sharp contrast, Ap-2α is not expressed in the colliculus inferior but strongly expressed in the anterior dorsal midbrain, the colliculus superior, and the cerebellum (Fig. 2e). Strikingly, the remaining dorsal midbrain of Ap-2δ−/− animals is positive for Ap-2α indicating that indeed the complete colliculus inferior is lacking in Ap-2δ mutant mice (Fig. 2f). The lack of the posterior midbrain might be either due to a defect in its formation during development or due to degradation in the absence of Ap-2δ. To test whether Ap-2δ is involved in the formation of the posterior midbrain during development we performed whole-mount in situ hybridizations with a set of marker genes, which are known to play essential roles during midbrain-hindbrain organization [24], [25], [26], [27], [28], [29]. Marker genes included *Pax2*, *Pax5*, *Fgf8*, *En1*, and *Wnt1*. Since no aberrant expression of these marker genes can be observed in 10 days old Ap-2δ-deficient embryos compared to control embryos, we concluded that Ap-2δ is either not required for early midbrain development or its loss can be compensated by the other Ap-2 transcription factors at this early developmental stage (Figure S2).

### Apoptosis leads to loss of colliculus inferior in Ap-2δ-deficient mice

**Figure 3:**
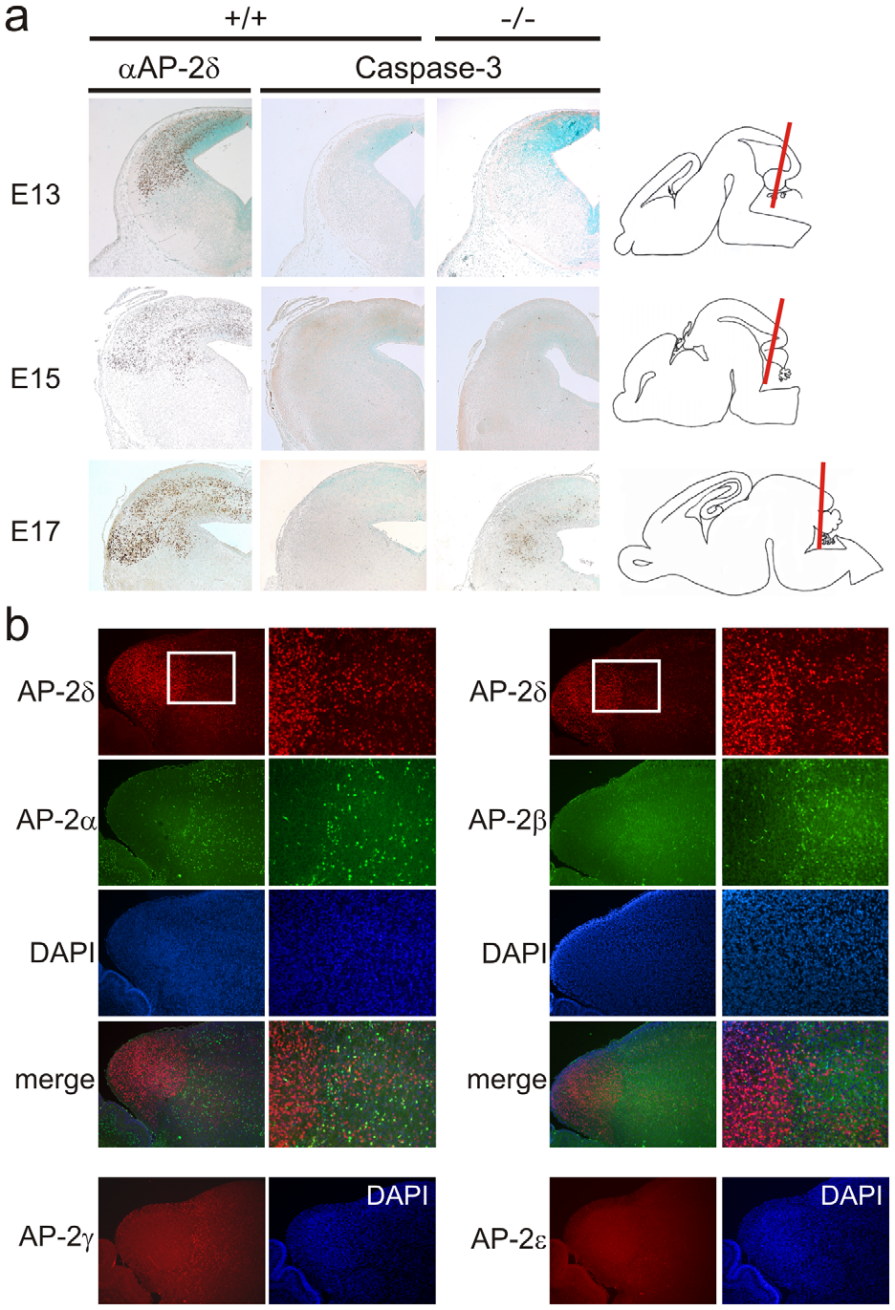
Massive apoptosis in colliculus inferior of Ap-2δ-deficient mice. (a) Immunohistological detection of Ap-2δ expression (left column) and apoptosis (middle and right column) with an activated caspase 3 antibody on coronal sections of embryonic brains of different developmental stages (E13-E17). Single apoptotic cells can be detected at E15 in the posterior midbrain of Ap-2δ-deficient mice (arrows), whereas massive apoptotic processes can be observed at E17. Right column: Indication of sectional plane through the brain. (b) Immunohistological characterization of Ap-2 isoforms on sagittal sections of midbrains from wild-type mice. Ap-2α, Ap-2β, Ap-2γ and Ap-2δ are expressed in the anterior midbrain (colliculus superior) of E17 embryos. Note that Ap-2δ is exclusively expressed in the colliculus inferior. Boxed areas are shown in the right panels. Expression of Ap-2ε was not detected in the midbrain at this developmental stage.

Ap-2 transcription factors are known to suppress apoptotic processes during embryogenesis [13]. Therefore, we investigated whether an increased cell death in the developing brain is responsible for the malformation. To this end, we examined coronal sections of embryonic brains at E13.5 to 17.5 by immunohistochemical staining for activated caspase 3 as a marker for programmed cell death. While no apoptosis can be detected in control and Ap-2δ mutant brains at E13.5, single apoptotic cells are labelled at E15.5 in the posterior midbrain of Ap-2δ-deficient mice. At E17.5 a dramatic increase in the number of apoptotic cells can be detected in the posterior midbrain of Ap-2δ mutants, while no cell death can be observed in sections from control embryos (Fig. 3a, middle and right column). To demonstrate that the apoptotic region co-localizes with Ap-2δ expression, consecutive wild-type sections were stained for Ap-2δ showing that indeed the region of apoptotic cell death overlaps with the expression of Ap-2δ in the posterior midbrain. These data indicate that Ap-2δ plays an essential role for the maintenance and further maturation of the posterior midbrain and loss of Ap-2δ results in the degradation of this structure due to massive apoptosis (Fig. 3a, right and left column).

### Ap-2 isoforms cannot compensate Ap-2δ deficiency in the posterior midbrain

Ap-2δ is also expressed in the anterior dorsal midbrain, the dorsal thalamus and cortex (Fig. 1a). In contrast to the massive apoptotic cell death in the posterior midbrain of Ap-2δ mutants, no apoptotic processes could be detected in these regions (data not shown) suggesting that Ap-2δ either plays a minor role for cell survival in these structures or other Ap-2 isoforms can compensate for its loss. Therefore, we analysed the expression of all Ap-2 isoforms in the brain of E17 embryos. Immunofluorescence stainings with Ap-2-specifc antibodies indicate Ap-2α, Ap-2β, Ap-2γ and Ap-2δ expression in the anterior dorsal midbrain but not in the posterior midbrain, the colliculus inferior (Fig. 3b). We could not detect any Ap-2ε expression in the midbrain at this developmental stage. Thus, Ap-2δ represents the only Ap-2 isoform expressed in the posterior midbrain at E17 and therefore its loss cannot be compensated by any other Ap-2 isoforms.

### Identification of Ap-2δ target genes in the developing posterior midbrain

**Figure 4:**
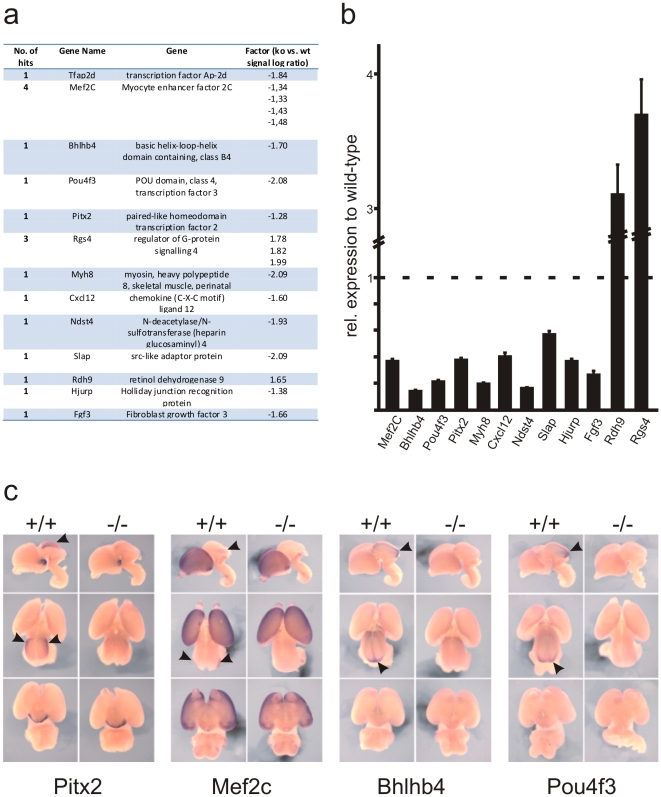
Identification of genes differentially expressed in the posterior midbrain of Ap-2δ wild-type and knockout mice. (a) List of differentially expressed genes in the posterior midbrain of E15 wild-type and Ap-2δ knockout embryos obtained from microarray analysis. (b) Validation of microarray data by real-time PCR. Relative expression levels of putative Ap-2δ target genes were determined from three independent qRT-PCR experiments, which were performed in triplicates. Data are presented as mean±SD. p-values (p<0.005) indicate significant differences from wild-type for each gene. (c) In situ hybridizations on E15 brains from wild-type and Ap-2δ-deficient embryos with specific cDNA probes of the transcription factors Pitx2, Mef2c, Bhlhb4 and Pou4f3. Shown are lateral (top), dorsal (middle) and ventral (below) views.

To understand Ap-2δ-dependent control of midbrain development, we identified Ap-2δ specific target genes that are involved in the formation of this brain structure. We isolated mRNA from wild-type and Ap-2δ−/− embryos at E15.5, shortly before tissue destruction becomes evident, and compared the expression patterns by microarray analysis. Using this approach we could identify 12 genes that showed a more than 2-fold difference in their expression levels between both samples (Fig. 4a). Quantitative RT-PCR analysis from total RNA samples isolated from the midbrains of E15.5 embryos verified their differential expression in Ap-2δ-deficient midbrain compared to wild-type tissue (Fig. 4b). While most genes showed reduced expression, two genes, Rdh9 and Rgs4, showed an increased expression in the absence of Ap-2δ. Notably, four transcription factors, Pitx2, Mef2c, Bhlhb4 and Pou4f3 were also identified as putative Ap-2δ target genes. Whole mount in situ hybridizations on E15.5 brains from control embryos verified that they are expressed in the midbrain. Importantly, loss of Ap-2δ abolished expression of these genes in the midbrain, while their expression patterns in other brain structures such as the forebrain and thalamus for Mef2c and Pitx2, respectively, were not altered (Fig. 4c).

### Ap-2δ binds to the Pou4f3 and Bhlhb4 promoter and activates transcription

**Figure 5:**
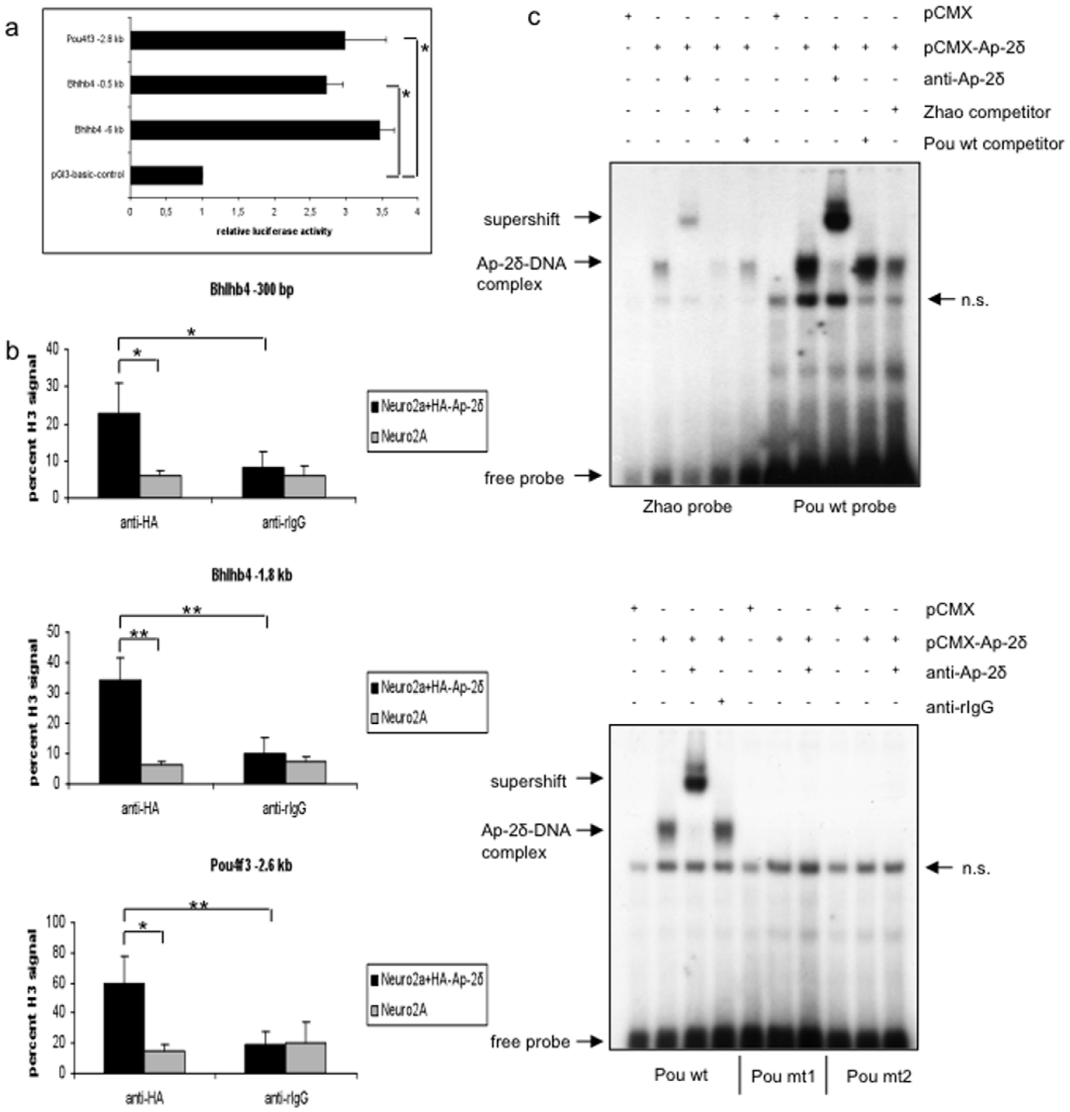
Ap-2δ binds to the Bhlhb4 and the Pou4f3 promoter. (a) Luc reporter plasmids were cotransfected with an Ap-2δ expression plasmid into Neuro2A cells. Ap-2δ activates native promoters of Bhlhb4 and Pou4f3 for more than 2.5-fold. The bars represent relative luciferase units normalized to total protein and show the mean ± s.e.m. of three independent transfections. Reporter-only transfections (pGl3-basic) were set at 1. p-values indicate significant differences from reporter-only transfected cells (student's t-test). (b) ChIP/qPCR assays in wild-type and HA-Ap-2δ transfected Neuro2a cells. (Top) Ap-2δ binds to the Bhlhb4 promoter 1.8 kb and 300 bp upstream of the TSS. (Bottom) Ap-2δ occupies the Pou4f3 promoter 2.8 kb upstream of the TSS. Bars represent mean abundance ± s.e.m. relative to histone H3 as determined from triplicate PCR measurements. p-values indicate significant differences from HA-Ap-2δ transfected Neuro2a cells precipitated with an anti-HA antibody compared to non-transfected Neuro2a cells or IgG treated lysates (student's-test) (c) (Top) EMSA was performed by incubating ^32^P-labeled, binding site-containing oligonucleotides with *in vitro*-translated Ap-2δ protein. The Ap-2δ/DNA complex is specifically supershifted with an α-Ap-2δ antibody and competed using an 80-fold excess of unlabeled oligonucleotide. (Bottom) EMSA was performed with an oligonucleotide containing the wildtype binding site of the Pou4f3 promoter (core sequence: GCC TGA GGG) or mutated forms (Pou mt1: GCC AGT GGG, Pou mt2: GCC GTT GGG). Upon mutation of the binding site, Ap-2δ binding is abolished.

To assess the transcriptional regulatory potential of Ap-2δ on Bhlhb4 and Pou4f3, we performed transient transfections with luciferase reporter constructs containing 6 kb and 500 bp fragments of the Bhlhb4 promoter (Bhlhb4–6 kb and Bhlhb4−0.5 kb, respectively) and a 2.6 kb fragment of the Pou4f3 promoter (Pou4f3−2.6 kb) upstream of the LUC reporter gene. Neuro2A cells endogenously express Ap-2δ and assumingly present a source of cofactors that might be indispensable for transactivation. Cotransfection of an Ap-2δ expression plasmid in Neuro2A cells induced transcriptional activation of all reporters for more than 2.5-fold which is within the activating potential of Ap-2 proteins (Fig. 5a).

### Ap-2δ occupies the Bhlhb4 and Pou4f3 promoter in vivo

To determine if Bhlhb4 and Pou4f3 are direct targets of Ap-2δ we performed chromatin immunoprecipitation (ChIP). We looked for evolutionary conserved Ap-2 binding sites on genomic loci of Bhlhb4 and Pou4f3 and found two putative binding sequences in the Bhlhb4 promoter and one sequence in the Pou4f3 promoter. ChIP was carried out on Neuro2a cells that had been transfected with an HA-tagged Ap-2δ expression plasmid or on untransfected cells. By this approach we could demonstrate that Ap-2δ occupies the promoter of Bhlhb4 at the previously identified conserved binding sites located −1.6 kb and −300 bp upstream of the transcriptional start site (TSS) (Fig. 5b upper panel). Furthermore, Ap-2δ is associated to the Pou4f3 promoter at a conserved recognition sequence 2.8 kb upstream of the TSS (Fig. 5b lower panel). Ap-2δ occupancy at these promoters could not be detected in untransfected cells or after immunoprecipitation with the control antibody (Fig. 5b).

### Direct binding of Ap-2δ to the Pou4f3 promoter

Ap-2 proteins bind to the evolutionary conserved binding sites GCCN_3/4_GGC or GCCN_3/4_GGG. Zhao et al. could show that Ap-2δ specifically binds to a sequence with TGA as the preferred central 3 bp spacer between the palindromic GCC motifs [8]. The sequence in the Pou4f3 promoter that we found to be occupied by Ap-2δ fulfills these criteria. We therefore investigated the direct binding of Ap-2δ protein to this sequence using electrophoretic mobility shift assays. *In vitro* translated Ap-2δ protein formed protein/DNA complexes with both oligonucleotides containing either the optimized Ap-2 binding site from Zhao et al. or the native binding site of the Pou4f3 promoter (Fig. 5c top, lanes 2 and 7). The complexes were supershifted by an α-Ap-2δ antibody but not an unrelated rIgG antibody (Fig. 5c top, lanes 3 and 8; Fig. 5c bottom, lanes 3 and 4). The retarded Ap-2δ/DNA complex is competed by an 80-fold molar excess of unlabeled oligo (lanes 4 and 5; 9 and 10). To analyze specificity of binding we introduced two mutations in the central 3 bp spacer. Mutation of the consensus sequence completely abolished Ap-2δ binding (Fig. 5c bottom). These data indicate that Ap-2δ binds to the Pou4f3 promoter in a specific manner.

### Loss of Ap-2δ results in downregulation of Pou4f3 target genes

Since loss of Ap-2δ leads to a significant downregulation of Pou4f3 transcription, we hypothesized that Ap-2δ deficiency might also have an effect on Pou4f3 target genes [30], [31]. Therefore, we quantified steady-state mRNA levels of Pou4f3-regulated genes in posterior midbrain samples of wild-type and Ap-2δ−/− mice. Pou4f3 transcript levels in Ap-2δ-deficient samples were reduced to less than 20% of wild-type levels. Consistently, we also observed a significant downregulation of Bdnf and Calbindin2, two Pou4f3 target genes; however, the expression of Lhx3, another Pou4f3 target gene, is not altered in Ap-2δ-deficient posterior midbrains (Figure S3).

### Electrophysiological recordings from neocortical neurons of Ap-2δ−/− mice

**Figure 6:**
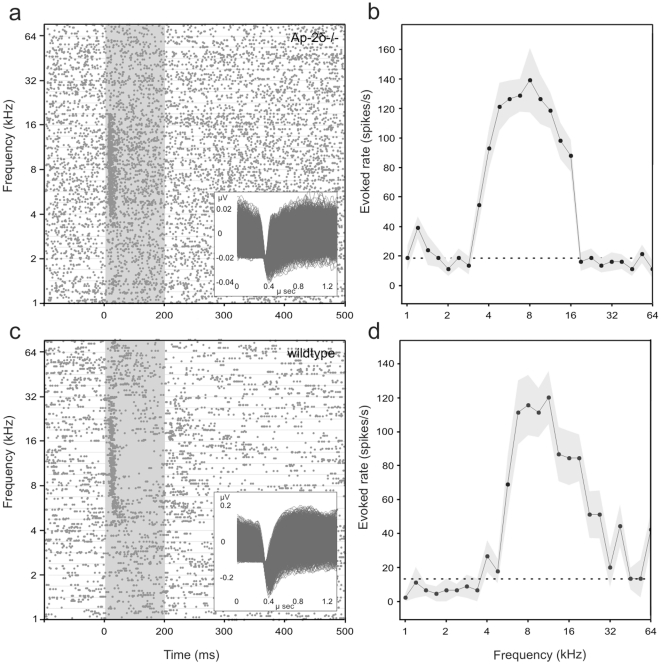
Comparison of neuronal response to tone stimulation from the neocortex of an Ap-2δ−/− and a wild-type mouse. (a) Raster plot from an Ap-2δ−/− mouse showing action potential (dots) of a small group of neurons that responded with an increased firing rate to the onset of tones of frequencies between 3.4 and 16 kHz. Each horizontal row of dots represents a trial with 15 trials per presented frequency (y-axis). The grey area covers the stimulus duration. The inset shows the spike waveforms of the recording. (b) The tone-evoked spike rate from (a) is plotted as a function of the stimulus frequency. Grey shade shows the standard error of the mean of the evoked rate, the dashed line the level of the spontaneous rate. The tone-evoked rate of the neuronal response is significantly higher than the spontaneous rate indicating auditory activity in the cortex. (c, d) Raster-plot (c) and rate function (d) from a wildtype mouse showing increased firing rate to the tone onset in a similar frequency range (4 to 38 kHz) as in Ap-2δ−/− mouse.

Since the colliculus inferior represents a central unit within the auditory pathway, we tested whether its absence impairs the transfer of auditory signals from the brainstem towards the neocortex in Ap-2δ−/− mice. Neurons in the caudal temporal cortex of two tested Ap-2δ−/− animals showed responses to tone bursts despite absence of the colliculus inferior. The example of a multi-unit recording in the raster-plot (Fig. 6a) shows a phasic response to tones in a limited frequency range. Fig. 6b clearly indicates an increased response rate in a frequency range between 3.4 and 16 kHz. The other recordings also showed phasic responses to tones tuned to certain frequency ranges altogether covering the range from 2.8 to 32 kHz. These data indicate that strong auditory responses are present at the neocortical level. For comparison a raster-plot of a multi-unit recording from a wild-type animal is shown in Fig. 6c, d. Responses from wild-type neurons also cover a certain frequency range (in this case 4 to 38 kHz) and are often phasic responses. Spike waveforms are shown in the insets (Fig. 6 a, c).

## Discussion

In this study, we analyzed the *in vivo* function of Ap-2δ by targeted inactivation of the *Tcfap2d* gene in mice. Using brain tissue from control and mutant mice we were able to compare their transcriptomes and identify Ap-2δ regulated genes. Ap-2 transcription factors have been found to be expressed in ectodermal, neuroectodermal and mesodermal tisses, where they are involved in controlling various developmental processes. In contrast to the broad expression patterns of Ap-2α to γ, the last two members of the Ap-2 transcription family, Ap-2δ and ε, show an almost neuronal specific expression pattern. Interestingly, all five members of the Ap-2 family are expressed in the roof of the midbrain during development. Ap-2δ, however, shows a highly dynamic expression pattern, in which it becomes enriched in the posterior midbrain and declines in the anterior midbrain. A similar expression pattern has not been described for any other Ap-2 transcription factor. Furthermore, beside its strong presence in the posterior dorsal midbrain, none of the other Ap-2 isoforms were found to be expressed there at the newborn stage, suggesting that Ap-2δ plays a pivotal role in this particular brain region. In line with this hypothesis, inactivation of Ap-2δ results in the degradation of the posterior midbrain and the absence of the colliculus inferior. Obviously, the initial organization and expansion of cells of the midbrain/hindbrain region is not affected by the absence of Ap-2δ as the expression of a number of marker genes did not show any alterations, although Ap-2δ is already expressed at this developmental stage. This suggests that the early midbrain formation is not dependent on Ap-2δ or its function is redundant, possibly through compensation by the expression of the other Ap-2 isoforms. However, massive apoptotic cell death occurs at the end of embryogenesis when Ap-2δ remains as the only expressed Ap-2 isoform.

Ap-2 proteins are involved in the suppression of apoptotic cell death in multiple developmental processes. Ap-2α to γ knockout mice reveal apoptotic processes at the main expression loci when compensation by other Ap-2 proteins was not given. Ap-2α mutant mice exhibited enhanced apoptosis in the anterior mid- and hindbrain and in the proximal mesenchyme of the first branchial arch [10]. We have demonstrated before that a decrease in expression of the anti-apoptotic genes bcl-X_L_, bcl-w and bcl-2 preceded massive apoptotic cell death in kidneys of Ap-2β-deficient mice [13]. Furthermore, cultured trophoblast cells of Ap-2γ-deficient blastocysts showed signs of morphological and apototic changes as well as a reduced expression of adenosine deaminase (ADA) [15]. These data show an essential role for Ap-2 transcription factors for the differentiation and survival of various different cell types.

Beside its role in suppressing cell death, Ap-2 transcription factors are key molecules that control cell differentiation by specifying target gene expression patterns. Chen et al. [32] reported that Ap-2 together with Sp-1 mediated differentiation by regulating K3 keratin transcription. A low Sp1/Ap-2 ratio accounted for suppression of K3 in undifferentiated basal cells whereas a high Sp1/Ap-2 ratio accounted for activation of K3 in differentiated corneal epithelial cells [32]. To analyse which target genes are controlled by Ap-2δ in the posterior midbrain we performed microarray analysis. Among the 12 putative targets four transcription factors were found which showed significant reduction in their expression. Whole mount in situ hybridization verified that Pitx2, Mef2c, Bhlhb4 and Pou4f3 are expressed in the midbrain of wild-type mice, but upon deletion of Ap-2δ the expression of these transcription factors was not detectable anymore in this brain structure. These data indicate that Ap-2δ is a central, higher order transcription factor that controls neuronal differentiation by controlling other transcription factors.

Functional classification of our genes downregulated in the absence of Ap-2δ revealed also genes involved in immunoregulation and possibly cell migration (Cxcl12 and Slap). Furthermore, genes controlling muscle development and contraction (Mef2c, Myh8), signal transduction (Rgs4) and metabolism (Ndst4, Rdh9) were found to be differentially expressed. Ap-2δ ablation also resulted in the significant downregulation of Pitx2 in the posterior midbrain. Notably, the promoter of the Pitx2 isoform, Pitx2c, is occupied and activated by Cited2 and Ap-2α in the heart [33]. So Pitx2c is a target of Ap-2α in the heart, however, which Pitx2 isoform is regulated by Ap-2δ in the midbrain remains to be analyzed.

Our *in vivo* Ap-2δ target gene screen was conducted on posterior midbrain samples of Ap-2δ-deficient animals. In contrast, previous studies analyzed Ap-2δ target gene expression after exogenous expression or siRNA-mediated knockdown of Ap-2δ in AD293 or Neuro2a cells, respectively [34], [35]. Our approach was performed to directly identify Ap-2δ target genes *in vivo*, which was also verified by qRT-PCR and in situ hybridizations, however, it is limited to genes expressed in the posterior midbrain.

Importantly, our data clearly show that Ap-2δ physically occupies in a sequence-specific manner an Ap-2-binding site located in the Pou4f3 promoter. Mutation of the binding site completely abolished Ap-2δ binding indicating high specificity. Pou4f3 plays an essential role in cell maturation and survival of cochlear hair cells in inner ear sensory epithelia. Pou4f3−/− mice suffer from defective inner ear function as a result of complete depletion of hair cells with subsequent loss of sensory neurons [36], [37]. Mutations of the POU4F3 gene have been related to autosomal dominant nonsyndromic hearing impairment DFNA15 in humans [38]. It remains to be shown, whether Ap-2δ is expressed in inner ear hair cells to control Pou4f3 expression.

The central auditory system processes the sound information from the inner ear via various nuclei in the brainstem towards the inferior colliculus (IC) of the midbrain. The central nucleus of the IC represents a relay station and sends the information to the thalamus. In mammals the auditory information is further processed in the medial geniculate body (MGB) of the thalamus, which sends projections to the auditory cortex. Interestingly, these auditory connections are already developed in mice before the onset of hearing indicating that molecular genetic clues determine the nuclear connections [39]. Although the colliculus inferior is a central unit of the auditory pathway [40], sounds evoked cortical neuronal responses in Ap-2δ−/− mice missing this nucleus. Our results demonstrate that tone-evoked responses occurred in temporal patterns and frequency tuning very similar to those recorded in the auditory cortex of wild-type mice under the same anesthetic conditions [41] [42]. Thus it seems that at least some higher auditory function beyond lower brainstem reflexes is preserved in Ap-2δ−/− mice. The fact that Ap-2δ−/− mice are able to process sounds at the neocortical level suggests that substantial and still to be determined, compensatory mechanisms during development must take place, e.g. bypassing the colliculus inferior in the ascending auditory pathways to the cortex and substituting its functional role. Alternatively, an auditory pathway bypassing the inferior colliculus has been described in bats and cats. This pathway visualized in the mustache bat originates in the nucleus of the central acoustic tract (NCAT), a group of large cells near the superior olive of the lower brainstem, ascends medial to the lateral lemniscus, and courses beneath the inferior colliculus and medial to the brachium of the inferior colliculus to reach the deep layers of the superior colliculus and the suprageniculate nucleus [43] [44]. The central acoustic tract might explain the simple response to individual tones in the auditory cortex in Ap-2δ-deficient mice. However, in which regard more complex processing tasks are affected in the absence of the inferior colliculus still needs to be determined. Accordingly, auditory function should be further analyzed using auditory discrimination learning tests. The Ap-2δ−/− mouse provides an excellent animal model to study higher auditory functions based on neuronal plasticity in the absence of the colliculus inferior. In addition, we and recently published ISH in Eurexpress could show that besides the prominent expression of Ap-2δ in the posterior midbrain, Ap-2δ is also expressed in the diencephalon, possibly the medial geniculate body (MGB), at moderate levels (www.eurexpress.org; assay ID: euxassay_007907). As already indicated,,the MGB is a thalamic relay station between the inferior colliculus and the auditory cortex. Therefore, further studies have to be conducted to study thalamocortical connections in Ap-2δ knockout mice.

In summary, we here provide evidence that Ap-2δ is essential for the suppression of apoptotic cell death in the posterior midbrain as depletion of Ap-2δ results in the loss of the colliculus inferior. Furthermore, we identify Bhlhb4 and Pou4f3 along with a set of other candidates as de novo Ap-2δ target genes and thus, our data define molecular mechanisms controlling development of the posterior midbrain by Ap-2δ.

## Supporting Information

Figure S1 Characterization of an Ap-2δ-specific polyclonal antibody raised against an N-terminal peptide. (a) Western blot analysis of protein extracts of HepG2 cells transfected with expression plasmids of all Ap-2 isoforms. The antibody specifically recognizes the 50 kDa Ap-2δ protein in cells transfected with an Ap-2δ expression plasmid without any cross reactivity with other Ap-2 isoforms. Anti-tubulin staining indicates equal loading. (b) Immunofluorescence stainings of NIH-3T3 cells co-transfected with an Ap-2δ expression plasmid and an EGFP-containing plasmid. EGFP-positive cells showed a nuclear staining after Ap-2δ-specific antibody incubation. (c) Whole mount in situ hybridization with an Ap-2δ-specific probe on E12.5 old embryos maps Ap-2δ expression to the midbrain (arrow) and the dorsal thalamus (arrowhead). (d) Immunohistological staining using the Ap-2δ antibody on sagittal sections of E12.5 old embryos detects Ap-2δ protein in the midbrain (arrow) and dorsal thalamus (arrowhead).
(TIF)

Figure S2 No defect in initial organization of midbrain. Whole mount in situ hybridization of 10 days old wild-type and Ap-2δ-deficient embryos with specific digoxigenin-labeled probes of marker genes characterizing midbrain-hindbrain organization during embryogenesis. The non-aberrant expression pattern of marker genes between wild-type and Ap-2δ-specific embryos indicates correct initiation of mid- and hindbrain formation.
(TIF)

Figure S3 Real-time qPCR analysis on Pou4f3-regulated genes after knockout of Ap-2δ. Total RNA was extracted from the posterior midbrain of wild-type and Ap-2δ knockout mice at E15. Expression levels were normalized to HPRT. Note that Ap-2δ knockout led to a significant decrease of Bdnf and Calb2, whereas steady-state level of Lhx3 is not affected by the loss of Ap-2δ. Statistical significance was calculated using the Student's t-test. A p-value less than 0.05 was considered to be significant. (*  =  p<0.05; **  =  p<0.005; ***  =  p<0.0005)
(TIF)
