# Supplementary figures and images for: AP-2δ Is a Crucial Transcriptional Regulator of the Posterior Midbrain
Source: PLoS One. 2011 Aug 9;6(8):e23483. doi: 10.1371/journal.pone.0023483 (PMC3153493; doi:10.1371/journal.pone.0023483)

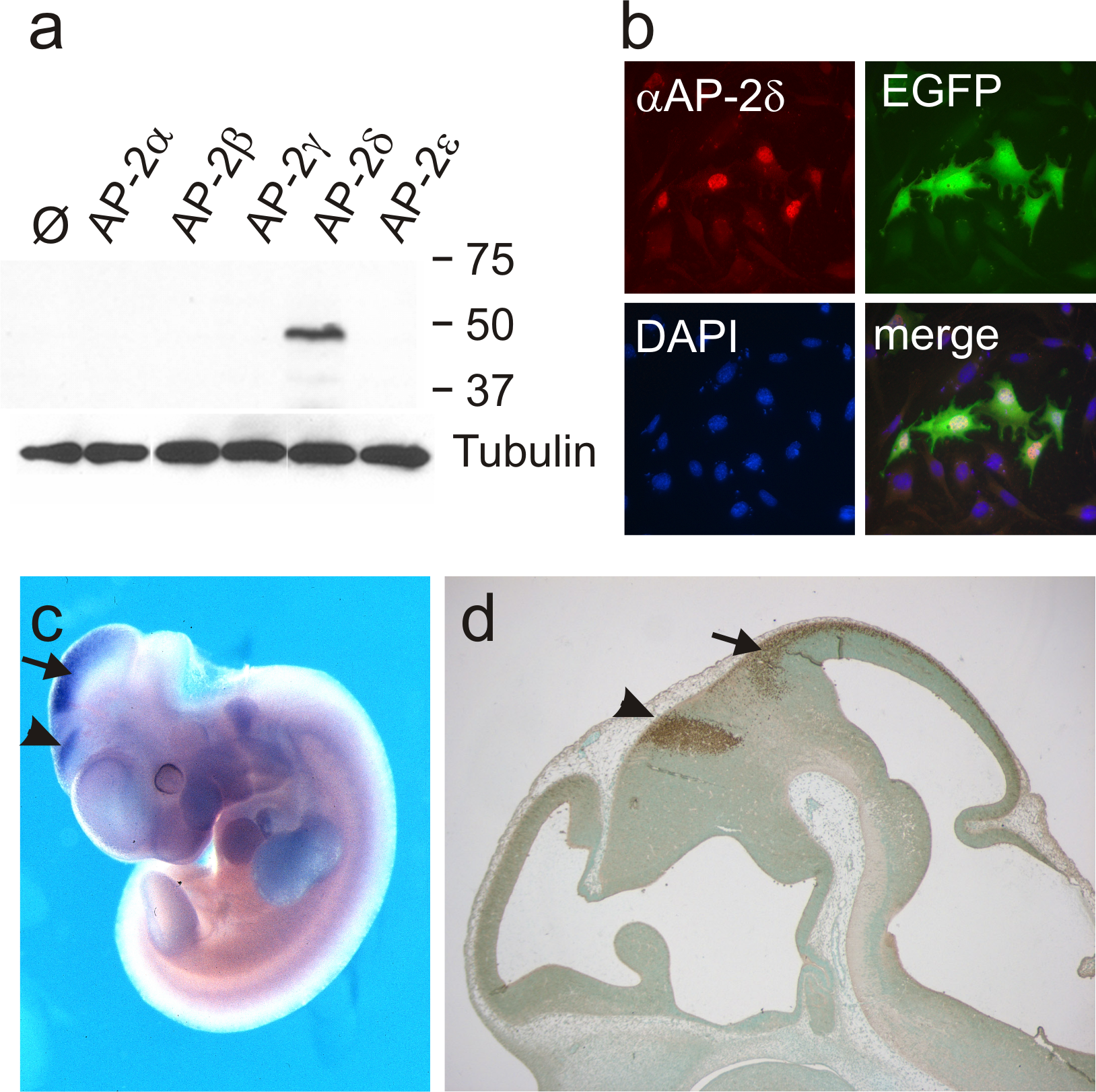

Supplement: Figure S1 — Characterization of an Ap-2δ-specific polyclonal antibody raised against an N-terminal peptide. (a) Western blot analysis of protein extracts of HepG2 cells transfected with expression plasmids of all Ap-2 isoforms. The antibody specifically recognizes the 50 kDa Ap-2δ protein in cells transfected with an Ap-2δ expression plasmid without any cross reactivity with other Ap-2 isoforms. Anti-tubulin staining indicates equal loading. (b) Immunofluorescence stainings of NIH-3T3 cells co-transfected with an Ap-2δ expression plasmid and an EGFP-containing plasmid. EGFP-positive cells showed a nuclear staining after Ap-2δ-specific antibody incubation. (c) Whole mount in situ hybridization with an Ap-2δ-specific probe on E12.5 old embryos maps Ap-2δ expression to the midbrain (arrow) and the dorsal thalamus (arrowhead). (d) Immunohistological staining using the Ap-2δ antibody on sagittal sections of E12.5 old embryos detects Ap-2δ protein in the midbrain (arrow) and dorsal thalamus (arrowhead). (TIF) [file pone.0023483.s001.tif]

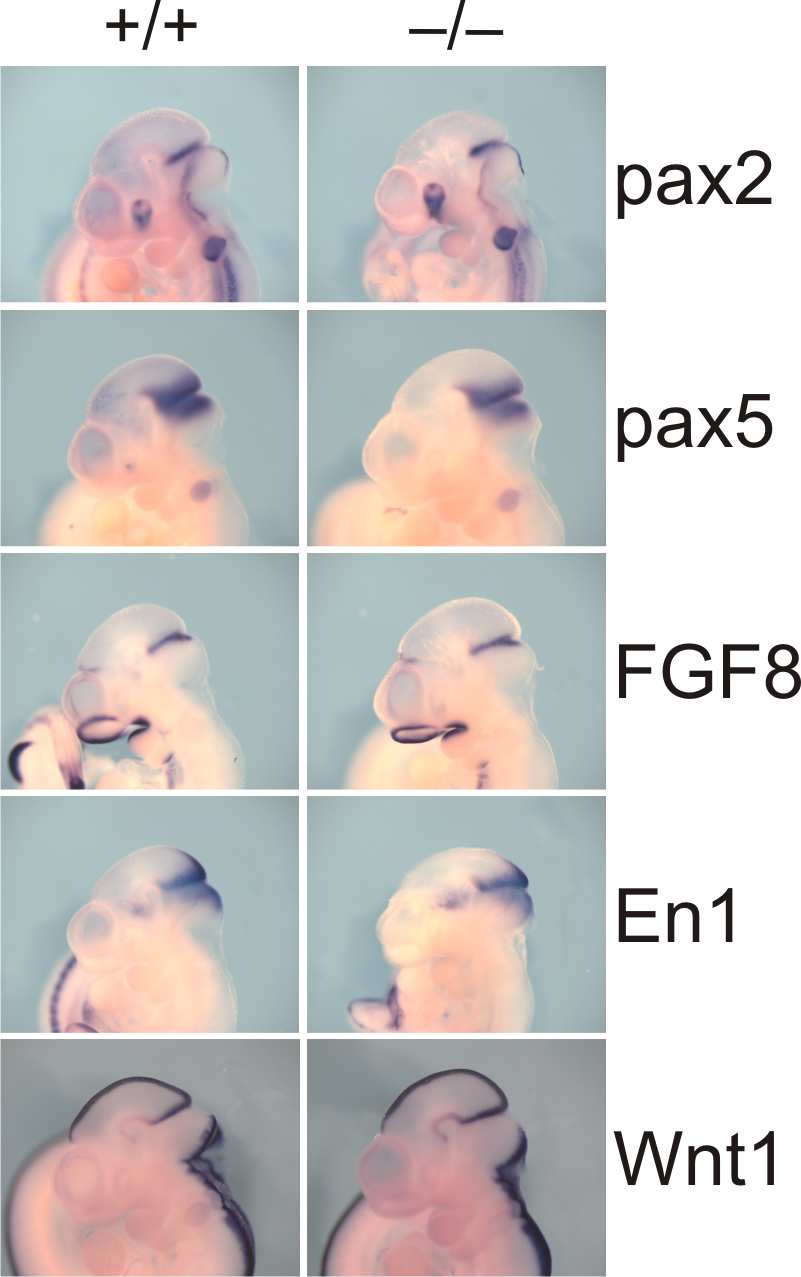

Supplement: Figure S2 — No defect in initial organization of midbrain. Whole mount in situ hybridization of 10 days old wild-type and Ap-2δ-deficient embryos with specific digoxigenin-labeled probes of marker genes characterizing midbrain-hindbrain organization during embryogenesis. The non-aberrant expression pattern of marker genes between wild-type and Ap-2δ-specific embryos indicates correct initiation of mid- and hindbrain formation. (TIF) [file pone.0023483.s002.tif]

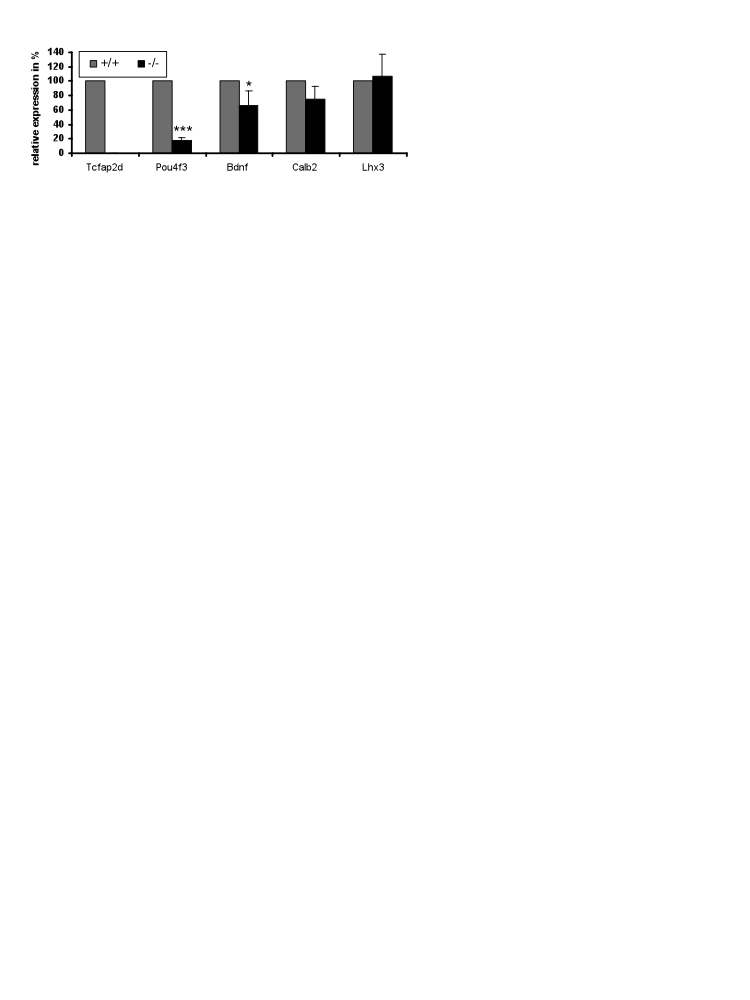

Supplement: Figure S3 — Real-time qPCR analysis on Pou4f3-regulated genes after knockout of Ap-2δ. Total RNA was extracted from the posterior midbrain of wild-type and Ap-2δ knockout mice at E15. Expression levels were normalized to HPRT. Note that Ap-2δ knockout led to a significant decrease of Bdnf and Calb2, whereas steady-state level of Lhx3 is not affected by the loss of Ap-2δ. Statistical significance was calculated using the Student's t-test. A p-value less than 0.05 was considered to be significant. (* = p<0.05; ** = p<0.005; *** = p<0.0005) (TIF) [file pone.0023483.s003.tif]
